# Effects of a multidisciplinary intervention to promote physical activity in patients with stroke undergoing rehabilitation: study protocol for the ActivePAS pilot randomised controlled trial

**DOI:** 10.1136/bmjsem-2022-001401

**Published:** 2022-10-25

**Authors:** Masashi Kanai, Masafumi Nozoe, Takuro Ohtsubo, Katsuhiro Ueno, Mai Nakayama, Masashi Yamashita, Kentaro Kamiya

**Affiliations:** 1 Department of Physical Therapy, Faculty of Nursing and Rehabilitation, Konan Women's University, Kobe, Japan; 2 Department of Rehabilitation, Nishi-Kinen Port Island Rehabilitation Hospital, Kobe, Japan; 3 Department of Rehabilitation Sciences, Graduate School of Medical Sciences, Kitasato University, Sagamihara, Japan

**Keywords:** Physical activity, Health promotion, Rehabilitation, Intervention efficacy, Accelerometer

## Abstract

**Introduction:**

Physical activity after stroke is related to functional recovery and outcomes. To optimise physical activity adapted to a patient’s walking ability and characteristics, multidisciplinary support and interventions are required. The Activate Physical Activity for Stroke pilot randomised controlled trial aims to assess the safety and feasibility of a multidisciplinary intervention that promotes physical activity in patients who had a stroke undergoing rehabilitation.

**Methods and analysis:**

This single-centre, randomised controlled trial will enrol 32 patients who had a stroke undergoing rehabilitation. Patients who had a stroke with the ability to walk 50 m with at least hand assistance, regardless of the use of braces or walking aids, and aged≥20 years will be randomly allocated to a multidisciplinary intervention group or control group. Patients in the intervention group will receive instructions for the self-monitoring of hospitalised physical activity and support to promote physical activity by multidisciplinary staff. The primary outcome of the present study is the safety (adverse events) and feasibility (retention and completion rates) of the multidisciplinary intervention. We assess physical activity using a triaxial accelerometer (UW-204NFC, A&D Company) as one of the secondary outcomes.

**Ethics and dissemination:**

The present study has been approved by the Research Ethics Committee of Konan Women’s University and the Ethics Committee of Nishi-Kinen Port Island Rehabilitation Hospital. We will disseminate the results of the present study through a peer-reviewed manuscript and presentations at international conferences.

**Trial registration number:**

UMIN000046731.

**WHAT IS ALREADY KNOWN ON THIS TOPIC**

- In patients who had a stroke undergoing rehabilitation, promoting physical activity is positively associated with functional recovery and functional outcomes.
- However, the effectiveness of strategies to promote physical activity in patients who had a stroke undergoing rehabilitation has not yet been consistently reported, and thus, these strategies remain unestablished.

**WHAT THIS STUDY ADDS**

- The Activate Physical Activity for Stroke pilot randomised controlled trial will examine the safety and feasibility of a multidisciplinary intervention tailored to a patient’s individual characteristics that promotes physical activity in patients who had a stroke undergoing rehabilitation.
- The present study will include interventions based on comprehensive and multimodal instruction concepts, such as instructing the self-monitoring of physical activity, observing physical activity using multiperson monitor supporting, and encouraging physical activity by multidisciplinary staff.

**HOW THIS STUDY MIGHT AFFECT RESEARCH, PRACTICE OR POLICY**

- The results of the present study will provide preliminary data to test the efficacy of this intervention in a larger population.

## Introduction

Stroke is a leading cause of various and complex disabilities and mortality. Lower physical fitness due to poor cardiorespiratory fitness or muscle strength and other impairments, such as cognitive function and balance, interact to drive poststroke activity limitations.^1^ Activity limitations lead to a negative loop of lower physical activity and further declines in physical fitness.^1^ The promotion of physical activity and improvements in physical fitness may be needed to break this negative loop. However, patients who had a stroke tend to be inactive and sedentary during hospitalisation.^2–4^ This has been reported in both acute and rehabilitation hospitals.^5^ In patients who had a stroke undergoing rehabilitation, physical activity, such as daily step counts, wheelchair self-propulsion distance, and intensity-based physical activity, has been associated with mobility following discharge and functional recovery and outcomes during hospitalisation.^6–8^ Therefore, strategies to promote physical activity from the early stages of hospitalisation may need to be implemented regardless of stroke severity or gait independence.

Self-management is essential in patients who had a stroke to promote physical activity^9 10^ and prevent undesirable health issues.^10 11^ A systematic review has shown that self-management interventions using behaviour change techniques can potentially engage patients who had a stroke in physical activity for behavioural changes.^12^ Regarding self-management interventions, a theory of self-efficacy^13^ is important.^14^ The self-efficacy of a particular activity increases due to a self-management intervention.^15^ Self-efficacy has been reported to improve along with an increase in physical activity with accelerometer-based feedback in hospitalised patients who had a stroke.^16^ Behavioural change techniques, such as goal setting, feedback and encouragement from medical staff or family members, have also been used for patients who had a stroke as a strategy to promote physical activity.^16–19^ However, several randomised controlled trials to promote physical activity using accelerometers or pedometers in patients who had a stroke undergoing rehabilitation showed no efficacy.^17–19^ Therefore, it is of limited relevance to patients who had a stroke and is difficult to generalise. Although inclusion criteria (particularly walking ability) vary among studies,^17–19^ comprehensive or multimodal interventions may be necessary for populations that include patients with low walking ability after stroke.

Yamada *et al* reported that the number of steps taken outside of rehabilitation was associated with the activities of daily living (ADL) in patients who had a stroke undergoing rehabilitation who had a high ability to walk.^20^ Shimizu *et al* found a relationship between light-intensity physical activity, such as standing, stepping, slow walking and ADL, during hospitalisation and improved gait independence in patients who had a stroke who required assistance or supervision in walking.^8^ They also suggested the importance of effectively using the time outside of rehabilitation and not depending solely on time in rehabilitation to promote gait independence.^8^ On the other hand, a systematic review of qualitative studies showed that patients who had a stroke undergoing inpatient rehabilitation valued physical activity and expressed a desire to practice more outside rehabilitation.^21^ During the time outside of rehabilitation, a therapist’s attention is not focused on the patient; therefore, comprehensive interventions and support for physical activity by multidisciplinary staff may be necessary. Furthermore, since this support and interventions are recommended to be personalised and multimodal,^22^ the walking ability and self-efficacy of a patient need to be considered. Therefore, we hypothesised that a multidisciplinary intervention tailored to a patient’s individual characteristics would effectively promote physical activity in patients who had a stroke undergoing rehabilitation.

### Objectives

The objective of the present study is to describe the study protocol for ‘a multidisciplinary intervention to Activate Physical Activity for Stroke—ActivePAS’. The primary objective of the ActivePAS pilot randomised controlled trial is to examine the safety and feasibility of the multidisciplinary intervention that promotes physical activity in patients who had a stroke undergoing rehabilitation. In addition, the present trial will assess the effects of a multidisciplinary intervention that promotes physical activity in these patients.

## Methods and analysis

This protocol is reported following Standard Protocol Items: Recommendations for Interventional Trials 2013 guidelines for clinical trial protocols.^23^

### Trial design

**Figure 1:**
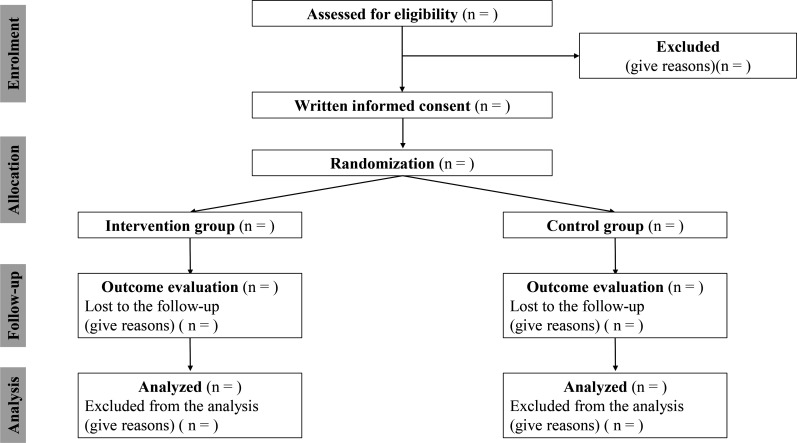
Study flow diagram.

This study is a single-centre, prospective, open-label, randomised controlled trial. An independent researcher not involved in enrolment or outcome measurements performed randomisation using a computer-generated 1:1 allocation sequence and permuted block size of 4. The flow chart of the present study is shown in figure 1.

### Study setting

This study will be conducted in a single convalescent hospital in Japan.

### Eligibility criteria

The inclusion and exclusion criteria for the present study are shown in box 1. Two or more physical therapists or physicians will assess patients’ eligibility to participate in the present study. We will include patients hospitalised for stroke with the ability to walk 50 m with at least hand assistance, regardless of the use of braces or walking aids, and aged ≥20 years. If a patient does not meet the above criteria at the time of admission, they will still be included in the present study if they can walk during hospitalisation. All patients will obtain informed consent before inclusion and randomisation (table 1).

Box 1 **Inclusion and exclusion criteria of the present study**

**Inclusion criteria**

1. Patients hospitalised for stroke
2. Patients with the ability to walk 50 m with at least hand assistance
3. Age≥20 years

**Exclusion criteria**

1. SAH
2. Stroke after cardiovascular surgery
3. Premorbid mRS≥3
4. Cognitive impairment (MMSE score<21)^33^
5. Patients who are expected to be hospitalised for less than 2 weeks
6. Patients who have neurological, orthopaedic, or medical diseases other than stroke that seriously affect physical activity
7. Patients who cannot give consent for research participation.

MMSE, Mini-Mental State Examination; mRS, modified Rankin Scale; SAH, subarachnoid haemorrhage.

**Table 1:** Summary of the study schedule

Time point	Study period			
	Baseline	Allocation	Intervention	
			Week 1	Week 4
Enrolment				
Demographic data	×			
Informed consent	×			
Allocation		×		
Interventions				
Intervention group				
Conventional rehabilitation	×		×	×
Multidisciplinary intervention			×	×
Self-monitoring of PA			×	×
Control group				
Conventional rehabilitation	×		×	×
Assessment				
Stroke severity				
NIHSS	×			
Motor dysfunction				
FMA-LE	×			
Safety			×	×
Feasibility	×		×	×
PA			×	×
Physical function				
Walking speed	×			×
SPPB	×			×
6MWD				×
Functional outcome				
FIM-M	×			×
Self-efficacy for PA	×			×

FIM-M, Functional Independence Measure for motor function; FMA-LE, Fugl-Meyer Assessment for lower extremity; 6MWD, 6-minute walking distance; NIHSS, National Institutes of Health Stroke Scale; PA, physical activity; SPPB, Short Physical Performance Battery.

The present study was registered at the University Hospital Medical Information Network Clinical Trial Registration before the enrolment of patients (ID: UMIN000046731). A flow chart of participants will be reported according to the Consolidated Standards of Reporting Trials guidelines (figure 1).

### Randomisation and blinding

Study enrolment will be implemented at a single convalescent hospital in Japan. Permuted block randomisation will be used, with a computerised random allocation sequence generated by an independent researcher throughout the study to facilitate data management. Randomisation is stratified by the ability or inability to walk independently. An answer form will be sent to the investigators, including a randomisation number.

Allocation results are disclosed to trial participants, outcome assessors and medical staff. Allocation results are not disclosed to the data analyst. To ensure that the statistical analysis is blind, the data analyst’s intervention/control group codes are masked until the data analysis is complete.

### Outcome measures

After enrolment, all patients wear an accelerometer on their waist belt 24 hours/day, except when bathing or changing clothes. We will use the average daily number of steps taken as an index of daily hospitalised physical activity. This will be measured by a UW-204NFC (A&D Company, Tokyo, Japan) three-dimensional accelerometer that calculates steps, total calories, calories burned and distance. Patients will wear the accelerometer for a minimum of 2 weeks and a maximum of 4 weeks.

**Figure 2:**
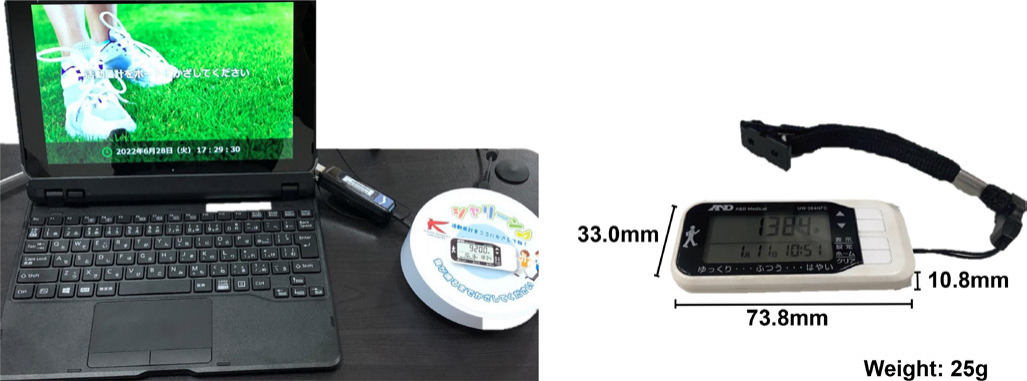
Multiperson physical activity monitoring system and a triaxial accelerometer.

**Figure 3:**
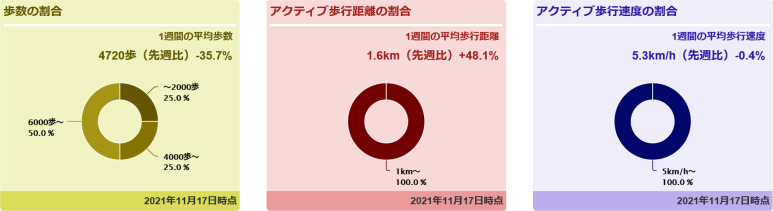
Visual feedback displayed on the multiperson monitor.

Daily hospitalised physical activity is sent to the multiperson physical activity monitoring system (MEDICA Cloud, Funabashi, Japan) through an offline connection. Medical staff, including a physician, nurse and physical therapist, will check physical activity through the monitor (figure 2). This multiperson monitor allows medical staff to observe not only a patient’s daily physical activity level but also provides a visual graph showing the degree of an increase or decrease from the previous week, a percentage breakdown of steps taken during the week, and the average walking speed when wearing the accelerometer (figure 3).

### Intervention

#### Conventional rehabilitation

Patients undergo a rehabilitation programme (≤3 hours/d) to improve ADL according to their functional disability impairment. These programmes include muscle strengthening training, balance training, standing training, walking training, ADL training, cognitive training, swallow training and speech training.

Outside of rehabilitation, patients can move around the ward and the bed based on an individualised multidisciplinary assessment of a patient’s mobility and cognitive abilities.

#### Intervention group

**Figure 4:**
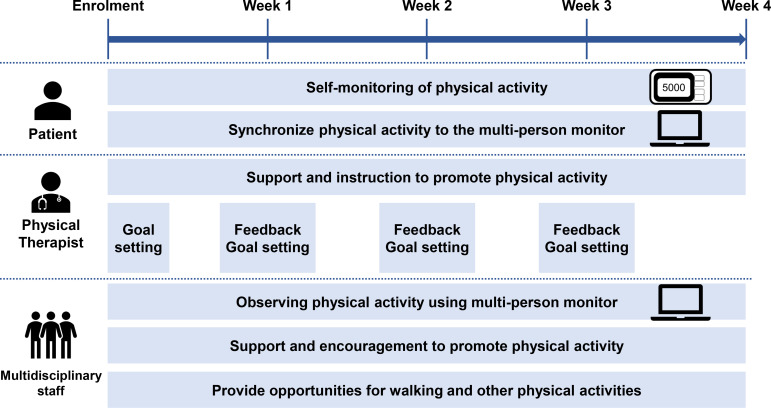
Overview of interventions.

An overview of the interventions is shown in figure 4. We will instruct patients in the intervention group to self-monitor their hospitalised physical activity. Specifically, we plan to use the method to self-monitor physical activity previously described by Kanai *et al* and Atkins *et al*.^16 19^ Patients in the intervention group will be asked by the physical therapist to record physical activity measured with the accelerometer on an exercise calendar at the end of the day. The patient and physical therapist collaboratively set a target number of steps per day, considering the patient’s walking ability and prestroke physical activity. By sharing these physical activity goals with multidisciplinary staff, we will encourage patients to promote physical activity outside rehabilitation. The standardised wording used by multidisciplinary staff to instruct patients is ‘try to walk more than you did last week’ and ‘keep self-monitoring your steps’. The physical therapist will provide visual feedback on changes in the number of steps taken over time and goal achievement to the patient every 7 days after study enrolment using the multiperson monitor. The physical therapist praises the patient if the patient achieves the physical activity target. If the patient does not achieve the physical activity target, the physical therapist will discuss a modified physical activity target with the patient by viewing the multiperson monitor display. With the instructions for self-monitoring physical activity and the multidisciplinary intervention, patients will be expected to improve their self-efficacy for physical activity (SEPA) and adopt other positive health behaviours.

We will instruct patients on the same self-monitoring methods, even if they need supervision or slight assistance to walk. However, we encourage walking and standing outside rehabilitation through multidisciplinary intervention to promote physical activity for these patients. For example, we provide patients with the opportunity to walk to the bathroom or dining room with medical staff at least once a day and stepping activities (such as stepping and forward stepping) at the bedside or by the chair. If these patients can walk independently during the intervention period, we will also consider setting a physical activity target as described above.

#### Control group

Patients in the control group only undergo the supervised rehabilitation programme. Patients in the control group will not be instructed on the methods to self-monitor physical activity and will be blinded to their physical activity. Medical staff are allowed to assist patients with walking and standing when requested. After the intervention period, a physical therapist will provide them with a visual report or feedback on their physical activity using the multiperson monitor.

### Primary outcome

The primary outcome of the present study is to examine the safety and feasibility of the multidisciplinary intervention. Outside of rehabilitation, we track and record all adverse events, such as falls, trauma requiring treatment, and neurological deterioration. An independent assessor (MN) will assess the number of adverse events during the intervention. To examine the feasibility of the multidisciplinary intervention, we will assess the retention rate (wearing the device during the intervention period) and the completion rate of the intervention. In addition, we will confirm the percentage of participants who meet the eligibility criteria throughout the study.

### Secondary outcomes

Secondary outcomes that are evaluated as exploratory are as follows:

1. Physical activity.
2. Difference in changes in walking speed between the baseline and after the intervention.
3. Difference in changes in Short Physical Performance Battery (SPPB) between the baseline and after the intervention.
4. Six-minute walking distance (6MWD).
5. Difference in changes in Functional Independence Measure motor function (FIM-M) between the baseline and after the intervention.
6. Differences in SEPA changes between the baseline and after the intervention.

We define physical activity as the number of steps taken during the last 7 consecutive days of the intervention period, which is planned to take 4 weeks. Even if patients are discharged within 4 weeks, we will use the number of steps taken in the last 7 days as the outcome for both the intervention and control groups as long as the patient has been wearing the accelerometer for at least 2 weeks. Physical activity is not blinded to the assessors because the accelerometer’s data synchronises with the multiperson monitor.

A baseline assessment is performed within 1 week of admission. Patients who can walk and become eligible for the study sometime after hospitalisation will undergo a baseline assessment on enrolment. A physical therapist will assess physical function, FIM-M, and SEPA. Comfortable walking speed is measured at 10 m along a 16-m walkway. A walking aid may be used if necessary at the time of the measurement. SPPB is an objective tool for evaluating lower extremity physical function.^24^ 6MWD is a submaximal exercise test used to assess functional capacity that will be performed according to the American Thoracic Society guidelines in a 30-m long straight corridor under the guidance of a physical therapist.^25^ Patients are allowed to use their usual assistive devices, and intermittent assistance for fall prevention will be provided as necessary.

FIM is one of the most common measurement tools for ADL.^26^ FIM-M has 13 subitems, and tasks are rated on a seven-point ordinal scale that ranges from requiring total assistance to complete independence. Walk subitem scores of FIM-M at baseline will be used for the allocation described above. Specifically, we stratify patients with walk subitems scores of 6 (modified independence) and 7 (complete independence) as the ability to walk, and those with scores of 4 (minimal assistance) and 5 (supervision) as an inability to walk. Physical or occupational therapists evaluate the functional status of all patients using this tool as part of the rehabilitation protocol during hospitalisation.

SEPA is measured with the Japanese version of the SEPA scale with validated reliability.^27^ This assessment was developed based on the study by Ewart *et al*.^28^ The measure consists of the following four subscales: the domain of walking, stair climbing, weight lifting and push-off, and subjects are asked to answer the degree of confidence for the activity load class. In the present study, we will use the walking domain as the SEPA index. SEPA scores range between 0 and 100, with lower scores indicating a poor level of SEPA and higher scores indicating a better level of SEPA.

We will assess baseline neurological deficits and motor dysfunctions as adjustment factors in statistical analysis. The former will be assessed by the National Institutes of Health Stroke Scale (NIHSS)^29^ and the latter by the Fugl-Meyer Assessment for lower extremity (FMA-LE).^30^ NIHSS is scored from 0 to 42 points, with higher scores indicating more severe symptoms. FMA-LE is scored from 0 (hemiplegia) to a maximum of 34 points. Seventeen items are included in lower limb FMA, and each item is scored on a three-point ordinal scale (zero=cannot perform, one=performs partially, and two=performs fully).

### Statistical analysis and sample size

We will perform intention-to-treat analyses. Results will be expressed as the mean±SE of the mean or the mean±SD for normally distributed data and as a median (25th–75th percentiles) for non-normally distributed data. The number of steps taken will be examined by an analysis of covariance (ANCOVA) with adjustments for baseline age, sex, NIHSS and FMA-LE. Other outcomes will be analysed by ANCOVA, an unpaired *t*-test, or the Mann-Whitney U test for continuous variables, and the χ^2^ test or Fisher’s exact test for categorical variables. A p value of <0.05 will be considered to indicate significance in all tests.

Since this is a pilot study, a formal power calculation was not performed. Based on the effect sizes of our previous studies on physical activity after stroke, one of the secondary outcomes, we will use a target sample size of 32 subjects as a guide.

## Discussion

The ActivePAS pilot randomised trial is a multidisciplinary intervention that includes comprehensive and multimodal instruction concepts. The present study plans to focus not only on patients who had a stroke who are walking independently but also on those who are not. We hypothesise that the multidisciplinary intervention will favour hospitalised physical activity in patients who had a stroke undergoing rehabilitation. Furthermore, we anticipate that the present study will be safe and feasible because it will use a multidisciplinary intervention tailored to the characteristics of each patient.

To our knowledge, previous studies have not found significant effects on physical activity in patients who had a stroke undergoing rehabilitation.^17–19^ The interventions in these studies incorporated the self-monitoring of physical activity, feedback and goal setting but may not have adequately considered patient characteristics. In addition, these studies were mainly performed by physical therapists.^17 18^ In the present study, the physical therapist, will be the main facilitator of physical activity interventions for patients; however, other medical staff may also monitor patients’ physical activity at any time with a multiperson monitor. Atkins reported that using a pedometer alone did not improve physical activity in a subacute stroke population due to a lack of individualised targets.^19^ They included patients who had a stroke with the ability to walk a minimum of 3 m with or without a gait aid or with or without the assistance of one person. To promote physical activity in non-ambulatory patients who had a stroke, it may be necessary for medical staff not simply to encourage walking but to provide opportunities for walking or standing actively. The present study anticipates promoting physical activity because it considers previous studies' limitations and provides a comprehensive, multimodal intervention based on patient characteristics and walking ability.

Physical activity in patients with stroke may be affected by their level of self-efficacy.^9 10 14 31 32^ In the present study, multidisciplinary staff will encourage walking outside of rehabilitation for patients with high walking function and self-efficacy and actively provide opportunities for walking and other physical activities outside of rehabilitation for patients with lower walking function and self-efficacy. Although self-efficacy differs among patients, self-efficacy is expected to improve with goal achievements or increased walking opportunities, resulting in increased physical activity.

The present study has some limitations. Since this is a randomised pilot study, it will only be conducted at a single centre in Japan. Therefore, caution needs to be applied to the results’ generalisability. Furthermore, since the present study implements a multidisciplinary intervention, much medical staff will know which group the patients are allocated to. This may result in bias. Although we include patients who had a stroke who can walk 50 m, the accelerometers used in this study have not yet been reported to be reliable for patients who had a stroke. In the present study, multidisciplinary staff encourage and provide opportunities to promote physical activity outside of rehabilitation; however, it is impossible to ascertain the extent to which they comply and how often the intervention is implemented. Therefore, we may not always be able to standardise and ensure the fidelity of our interventions.

## Conclusions

The ActivePAS pilot randomised controlled trial will assess the safety and feasibility of a multidisciplinary intervention to promote physical activity in patients who had a stroke undergoing rehabilitation. The results will provide a novel strategy for promoting physical activity in patients who had a stroke undergoing rehabilitation.

## Data Availability

Data sharing not applicable as no datasets generated and/or analysed for this study.
